# Negatively charged α-synuclein condensate modulates partitioning of molecules

**DOI:** 10.1016/j.jbc.2025.110530

**Published:** 2025-07-26

**Authors:** Qingqing Yang, Shunfa Chen, Pengfei Zhang, Zhonghua Lu, Shuwen Chang, Leo E. Wong

**Affiliations:** 1 Institute of Biomedical and Health Engineering, Shenzhen Institutes of Advanced Technology, Chinese Academy of Sciences, Shenzhen, China; 2 Shenzhen Key Laboratory for Molecular Biology of Neural Development, Shenzhen Technological Research Center for Primate Translational Medicine, Shenzhen-Hong Kong Institute of Brain Science, Shenzhen Institutes of Advanced Technology, Chinese Academy of Sciences, Shenzhen, China; 3 Guangdong Key Laboratory of Nanomedicine, CAS-HK Joint Lab for Biomaterials, CAS Key Laboratory of Biomedical Imaging Science and System, Shenzhen Engineering Laboratory of Nanomedicine and Nanoformulations, Institute of Biomedicine and Biotechnology, Shenzhen Institutes of Advanced Technology, Chinese Academy of Sciences, Shenzhen, China; 4 Institute of Brain Cognition and Brain Disease Institute, Shenzhen Institutes of Advanced Technology, Chinese Academy of Sciences, Shenzhen, China; 5 Shenzhen Neher Neural Plasticity Laboratory, Shenzhen-Hong Kong Institute of Brain Science-Shenzhen Fundamental Research Institutions, Shenzhen, China

**Keywords:** α-synuclein, biomolecular condensates, biophysics, electrostatics, intrinsically disordered protein, Parkinson disease

## Abstract

α-Synuclein (αSyn) aggregation *via* liquid–liquid phase separation (LLPS) has recently emerged as a crucial mechanism underlying amyloid fibril formation implicated in Parkinson’s disease. However, comprehensive investigation of the physicochemical properties of αSyn condensate remains incomplete. Here, we demonstrate that αSyn condensates exhibit a highly negative electrostatic potential, revealed by preferential enrichment of positively charged fluorophores or fluorophore-labeled αSyn. We further demonstrate that αSyn LLPS is regulated by a balance between self-association and electrostatic repulsion, with excess negative charge inhibiting phase separation. To study αSyn LLPS in a cellular context, we generated a stably transfected SH-SY5Y cell line that forms gel-like, nontoxic αSyn condensates upon differentiation. Most importantly, these intracellular condensates maintain a negative electrostatic potential, as evidenced by mEGFP probes systematically engineered with increasing positive charge. Our findings underscore the central role of electrostatic potential in modulating molecular partitioning within αSyn condensates and suggest that similar electrostatic principles may apply to other biomolecular condensates.

Parkinson’s disease (PD) is the second most prevalent neurodegenerative disease after Alzheimer’s disease (1). Although PD has a complex etiology, the synaptic protein α-synuclein (αSyn) remains a prime suspect in its pathogenesis. Notably, αSyn is a major component of Lewy bodies—the characteristic intracellular inclusions found in affected brain regions of PD patients (2, 3). In its monomeric form, αSyn is predominantly intrinsically disordered (4, 5, 6), but it also exists in various oligomeric and fibrillar forms that can exhibit pathogenic properties (7, 8, 9). While αSyn aggregation is widely believed to play a critical role in PD, a unified understanding of αSyn’s behavior *in vivo* during PD progression remains elusive (10, 11).

Recent studies have demonstrated that αSyn can undergo liquid–liquid phase separation (LLPS) (12), with phase-separated droplets maturing over days to form hydrogels that eventually nucleate amyloid fibril formation (13, 14). A similar mechanism, in which mutated proteins transition through a phase-separated state to promote aggregation, has been proposed for FUS (15, 16) and TDP-43 (17) in amyotrophic lateral sclerosis, as well as for tau (18, 19, 20) in Alzheimer's disease. These findings suggest that LLPS-promoted protein aggregation may represent a common pathological mechanism underlying multiple neurodegenerative diseases. This insight not only inspires novel therapeutic strategies targeting protein LLPS modulation (21) but also highlights the need for in-depth investigation of molecular partitioning mechanisms within these condensates (17).

αSyn is an acidic protein with a predicted net charge of approximately −9 at pH 7.4. Our study reveals that αSyn condensates formed through LLPS exhibit a strongly negative electrostatic potential, leading to differential partitioning of dye-labeled αSyn variants by up to 10-fold depending on the dye's net charge. Zeta potential measurements confirmed the formation of an electric double layer at the condensate surface. Using cyanine and riboflavin derivatives, we further demonstrate that small molecules partition into αSyn condensates in a charge-dependent manner. Interestingly, we found that αSyn LLPS propensity is governed by a balance between associative interactions and charge repulsion among αSyn molecules, with condensate properties being influenced by the incorporated dye's charge. Consistent with *in vitro* LLPS observations, intracellular αSyn condensates also displayed negative electrostatic potential. While the phenomenon of surface potential in biomolecular condensates has been recently reported (22, 23, 24), our work demonstrates the profound impact of condensate electrostatic potential on molecular partitioning. Furthermore, we establish a straightforward method for probing intracellular condensate electrostatic potentials.

## Results

### Distribution of dye-labeled αSyn is charge-dependent

**Figure 1:**
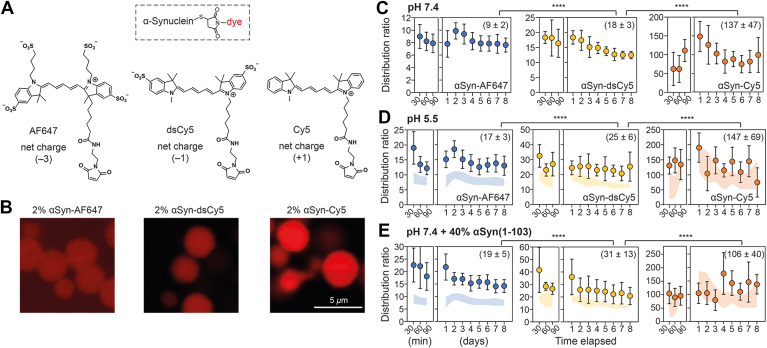
**Partitioning of αSyn labeled with tags of different charges.***A*, molecular structures of Alexa Fluor 647 C2 maleimide (AF647, net charge: −3), sulfo-Cy5 maleimide (dsCy5, −1), and Cy5 maleimide (Cy5, +1). Michael addition of the thiol group to maleimide results in a single maleimide-thiol conjugate between αSyn(A140C) and each respective dye. *B*, fluorescence images of condensates formed by 200 *μ*M αSyn doped with 2% molar equivalent of either AF647-, dsCy5-, or Cy5-labeled αSyn at pH 7.4. *C*–*E*, distribution ratios of AF647-, dsCy5-, and Cy5-labeled αSyn, respectively, at indicated time points post-LLPS in different buffer conditions: (*C*) pH 7.4 (25 mM Hepes, 150 mM NaCl, and 10% w/v PEG-8000), (*D*) pH 5.5 (25 mM MES, 150 mM NaCl, and 10% w/v PEG-8000), or (*E*) pH 7.4 as a mixture of 120 *μ*M full-length αSyn and 80 *μ*M αSyn(1-103). Data represent mean ± s.d. of 50 to 60 measurements from two independent samples. *Shaded areas* in (*D*) and (*E*) show mean ± s.d. from (*C*) for comparison. Average values from days 1 and 2 (d1 and d2) are shown in parentheses. Statistical significance was assessed by two-tailed *t* tests (∗∗∗∗ for *p* < 0.0001). αSyn, α-synuclein; LLPS, liquid–liquid phase separation; PEG, polyethylene glycol.

To determine whether the partitioning of dye-labeled αSyn is influenced by electric charge, we selected a series of cyanine fluorophores, *i.e.* AF647, dsCy5, and Cy5 with respective net charges of −3, −1, and +1, to label αSyn(A140C) *via* thiol-maleimide conjugation (Fig. 1*A*). The C-terminal alanine residue was chosen as the conjugation site to minimize disruption of αSyn’s ensemble structure and intermolecular interactions. Circular dichroism confirmed that all cyanine-labeled αSyn variants largely retained the secondary structure of native αSyn (Fig. S1).

Upon LLPS sample preparation (see Experimental Procedures and Fig. S2), sparse, small (<1–5 *μ*m), and highly mobile droplets formed immediately, as observed by fluorescence and differential interference contrast microscopy. Droplet size stabilized after 60 to 90 min, and distribution ratios of dye-labeled αSyn were quantified for up to 8 days post-LLPS. According to IUPAC recommendation (25), we defined the *distribution ratio* as the total concentration of dye-labeled αSyn (or organic fluorophore) within droplets relative to its concentration outside droplets, regardless of chemical form. This differs from the *partition coefficient*, which strictly refers to unionized species. At 37 °C and pH 7.4 (25 mM Hepes, 150 mM NaCl, and 10% w/v PEG-8000), partitioning of the three cyanine-labeled αSyn variants was distinct and correlated with tag charge (Fig. 1, *B* and *C*): αSyn-Cy5 had ≥ 10-fold greater enrichment in droplets *versus* αSyn-AF647, while αSyn-dsCy5 had double the distribution ratio of αSyn-AF647. This trend indicates the presence of a negative electrostatic potential within the droplets.

Assuming the difference in distribution ratios between αSyn-dsCy5 (relative charge = −1) and αSyn-AF647 (relative charge = −3) arises solely from electrostatic repulsion due to the two additional negative charges, we can estimate the electrostatic potential (*ψ*) of the condensates at pH 7.4 and 37 °C. First, the distribution ratio (*K*) can be used to calculate the generalized standard free energy of transfer (Δ*G*^tr^) *via* the relationship Δ*G*^tr^ = –*RT*ln*K*, where *R* is the gas constant (8.314 J/mol K) and *T* is the temperature in Kelvin (26, 27). From the distribution ratios of αSyn-AF647 and αSyn-dsCy5, we obtained Δ*G*^tr^ of −5.7 and −7.5 kJ/mol, respectively. The difference (ΔΔ*G*^tr^ = 1.8 kJ/mol) represents the electrostatic work (*U*_E_) required to move two additional negative charges (*z* = −2) into the condensate. Hence, the electrostatic potential of αSyn condensates at pH 7.4 was estimated to be −9.3 mV using the relationship *U*_E_ = *zFψ*, where *z* is the charge number and *F* is Faraday constant (96,485 C/mol). Based on the same principle, untagged αSyn (relative charge = 0) and αSyn-Cy5 (relative charge = +1) were expected to have a distribution ratio of 25 and 51, respectively. Since the measured distribution ratio of αSyn-Cy5 was more than 100 (Fig. 1*C*), the cyanine moiety of αSyn-Cy5 is likely to have additional interactions with αSyn condensate beyond the described electrostatic field (see below).

If the electrostatic potential of αSyn condensates were directly proportional to αSyn's predicted net charge (determined by its ionizable side chains), decreasing the protein's net negative charge should lead to increased enrichment of αSyn-AF647 and αSyn-dsCy5 while decreasing αSyn-Cy5 enrichment. We simulated this scenario under two conditions: (i) pH 5.5 buffer (25 mM MES, 150 mM NaCl, 10% w/v PEG-8000) and (ii) a mixture of full-length αSyn with C-terminally truncated αSyn(1-103) in pH 7.4 buffer. The predicted net charges under these conditions were −6.5 and −4.1, respectively, compared to −9.7 for αSyn at pH 7.4 (Fig. S3). Notably, αSyn(1-103) occurs physiologically and is found in Lewy bodies (28).

In both conditions, we observed increased distribution ratios for αSyn-AF647 [90% at pH 5.5; 110% in the αSyn(1-103) mixture] and αSyn-dsCy5 (40% and 70%, respectively) (Fig. 1, *D* and *E*). Interestingly, αSyn-Cy5 showed modest enrichment (7%) at pH 5.5 but slightly decreased in the αSyn(1-103) mixture. These results support the hypothesis that αSyn's net charge influences condensate electrostatic potential. However, the overall effect may also involve concurrent changes in the structural ensembles of αSyn under different conditions, as αSyn is known to adopt more compact, aggregation-prone conformations at lower pH (29, 30).

Since αSyn condensates exhibit maturation—characterized by progressively reduced internal diffusion and increased thioflavin T fluorescence (13, 14)—we further investigated the material state of our LLPS samples. Strikingly, αSyn condensates formed after 30 min of sample preparation showed minimal fluorescence recovery after photobleaching (Fig. S4*A*), suggesting that LLPS is followed by a sol-gel transition, a phenomenon previously reported for other LLPS-prone proteins like full-length TDP-43 (31). Consistent with a gel-like state, these αSyn condensates were resistant to disruption by 10% (w/v) 1,6-hexanediol (1,6-HD) (32) but could be dispersed by 1% (w/v) sodium dodecyl sulfate (SDS) (33) (a surfactants capable of combined hydrophobic and electrostatic disruption) (Fig. S4*B*). Finally, we monitored αSyn fibrillation *via* thioflavin T fluorescence and observed no significant fibril formation for up to 7 days post-LLPS (Fig. S4, *C* and *D*).

### Negative zeta potential of αSyn condensates

**Figure 2:**
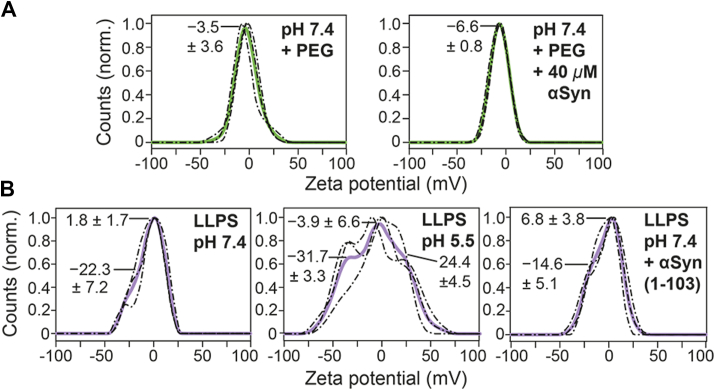
**Zeta potential of αSyn condensates.***A–B*, distribution of the particles’ zeta potential measured for (*A*) pH 7.4 LLPS buffer with and without 40 μM αSyn and (*B*) LLPS samples containing 200 μM αSyn under different conditions: pH 7.4, pH 5.5, and pH 7.4 mixed with 40% αSyn(1-103). *Solid curves* represent the average of triplicate measurements (*dashed curves*). The zeta potential values of the peaks (denoted by *straight lines*) were obtained by fitting the distributions to gaussian mixture models. αSyn, α-synuclein; LLPS, liquid–liquid phase separation.

Zeta potential is the electrical potential at the slipping plane of the double layer of dispersed particles (34). Given the evidence that αSyn condensates possess a negative electrostatic potential that responds to either pH changes or mixing with a truncated αSyn, we attempted to measure the zeta potential of the condensates formed under different conditions using a commercial instrument. Since the samples contained a substantial amount of polyethylene glycol (PEG), a single peak with a single-digit negative zeta potential was obtained for the samples without phase-separated αSyn (Fig. 2*A*). We later double-checked the identity of this peak in samples with either a higher concentration of PEG or PEG with a higher concentration of NaCl and still obtained a single-digit zeta potential (Fig. S5).

When the phase-separated samples were measured, an additional peak with lower amplitude was clearly observed on the left side of the more dominant peak attributed to PEG (Fig. 2*B*), supporting the presence of a negative zeta potential on the surface of the αSyn condensates. However, the broad distributions failed to provide quantitative information on any possible differences between the experimental conditions. We noticed that the resolution of the distribution depended on the maximum voltage applied. Due to the samples’ high conductivity, the maximum voltage was capped at 50 V during these measurements. In addition, high polydispersity in the zeta potential of biomolecular condensates (22) could also be a contributing factor. The conductivity of these samples, as well as the measured electrophoretic mobility, are presented in the Supporting Information (Table S1). Note that an unknown species with a positive zeta potential was also observed in the LLPS sample at pH 5.5, which might be an artifact caused by an electrode-analyte reaction.

### Net charge-dependent molecular partitioning into αSyn condensate

**Figure 3:**
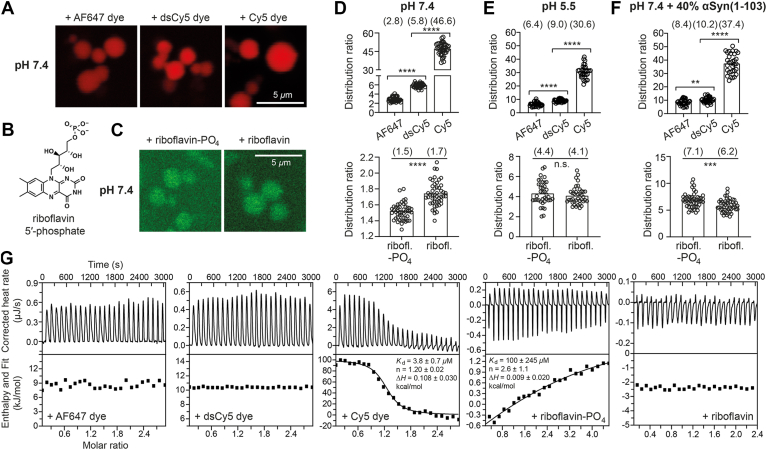
**Partitioning of fluorophores with different net charges.***A, C*, fluorescence images of αSyn condensates incubated with (*A*) 20 *μ*M AF647-, dsCy5-, and Cy5-maleimide and (*C*) 20 *μ*M riboflavin-5′-phosphate (riboflavin-PO_4_) and riboflavin, respectively, at pH 7.4 and day 1 (d1) post-LLPS. *B*, chemical structure of riboflavin-5′-phosphate (riboflavin-PO_4_). *D–F*, distribution ratios of the fluorophores (cyanines in *upper panel*; riboflavins in *lower panel*) in LLPS samples under different buffer conditions: pH 7.4 (cyanines, *n* = 40; riboflavins, *n* = 48), pH 5.5 (cyanines, *n* = 33; riboflavins, *n* = 39), and pH 7.4 mixed with 40% αSyn(1-103) (cyanines, *n* = 32; riboflavins, *n* = 48). Mean values are indicated in parentheses. Statistical significance was assessed by two-tailed *t* tests (n.s. for *p* > 0.05, ∗∗ for *p* < 0.01, ∗∗∗ for *p* < 0.001, ∗∗∗∗ for *p* < 0.0001). *G*, ITC thermograms and binding isotherms for titrations of the respective fluorophores into αSyn solution. αSyn, α-synuclein; ITC, isothermal titration calorimetry; LLPS, liquid–liquid phase separation.

Since electrostatic potential had a significant effect on the enrichment of cyanine-labeled αSyn, αSyn condensate most probably can modulate the partitioning of chemical compounds based on the same principle. As a proof of principle, we used directly the three cyanine-maleimide fluorophores that are structurally similar except for their net charges and tested them on the LLPS sample of wildtype αSyn, in which the thiol-maleimide reaction was absent. Being freely diffusing dyes, the cyanine-maleimide were partitioned surprisingly strongly into αSyn condensates but to different extents depending on their respective net charges (Fig. 3, *A* and *D*). From the respective distribution ratio of AF647 (2.8) and dsCy5 (5.8), we obtained their ΔΔ*G*^tr^ of −1.9 kJ/mol, which is very close to the value calculated for αSyn-AF647 and αSyn-dsCy5. This result strongly supports the conclusion that electrostatic repulsion plays a major role in the differential partitioning of the two cyanine-labeled αSyn. Similar to the case of αSyn-Cy5, Cy5-maleimide had a distribution ratio of 46.6, which is larger than the expected value of 12. This can be explained by the enhanced binding of Cy5-maleimide to αSyn due to the lack of sulfonate groups (Fig. 3*G*, third panel).

Likewise, we examined the partitioning behavior of two additional fluorophores: riboflavin and its phosphorylated derivative, riboflavin-5′-phosphate, which differ solely by a negatively charged phosphate group. As anticipated, riboflavin-5′-phosphate showed weaker condensate partitioning than riboflavin (Fig. 3, *B*–*D*, lower panel), further supporting our conclusion that negative charge antagonizes enrichment in αSyn condensates.

We next investigated how pH 5.5 and a mixture with αSyn(1-103) altered the partitioning of these fluorophores and cyanine derivatives. For the cyanine derivatives, the trends of changes closely matched those observed for dye-conjugated αSyn (Fig. 1, *C*–*E*): AF647- and dsCy5-maleimide became more enriched in condensates at pH 5.5, while Cy5-maleimide became less enriched (Fig. 3, *E* and *F*, upper panels). On the other hand, the distribution ratios of riboflavin-5′-phosphate and riboflavin equalized at pH 5.5 (Fig. 3*E*, lower panel), indicating either a reduction in condensate electrostatic potential or protonation of the phosphate group (p*K*_a_ ∼6.5). Lastly, riboflavin-5′-phosphate became more enriched than riboflavin in the condensates mixed with αSyn(1-103) (Fig. 3*F*, lower panel), which is also consistent with the presumption that these condensates have a lower electrostatic potential.

To investigate whether the differential partitioning can be accounted for by the binding affinity of the respective fluorophores to αSyn protein, we ran the isothermal titration calorimetry (ITC) experiments by titrating the fluorophores into αSyn solution at pH 7.4 (Fig. 3*G*). Both AF647- and dsCy5-maleimide exhibited negligible binding to αSyn, while Cy5-maleimide bound to αSyn with *K*_d_ of 3.8 ± 0.7 *μ*M, indicating that specific binding of Cy5-maleimide might contribute to its large distribution ratio in phase-separated αSyn (Fig. 3*D*, upper panel). On the other hand, riboflavin-5′-phosphate bound to αSyn with *K*_d_ of 100 ± 245 *μ*M, while riboflavin had negligible binding to αSyn. Even though riboflavin-5′-phosphate has a higher affinity to αSyn than riboflavin, it was less enriched in αSyn condensate at pH 7.4 (Fig. 3*D*, lower panel), indicating the condensate’s electrostatic field in action. In overall, these results support a positive correlation between the fluorophores’ net charge and their partitioning into αSyn condensate. This correlation is likely due to their interaction with the negative electrostatic potential within αSyn condensate, in addition to the partial contribution of their individual binding affinity to αSyn protein.

### *α*Syn LLPS is antagonized by excess negative charge

**Figure 4:**
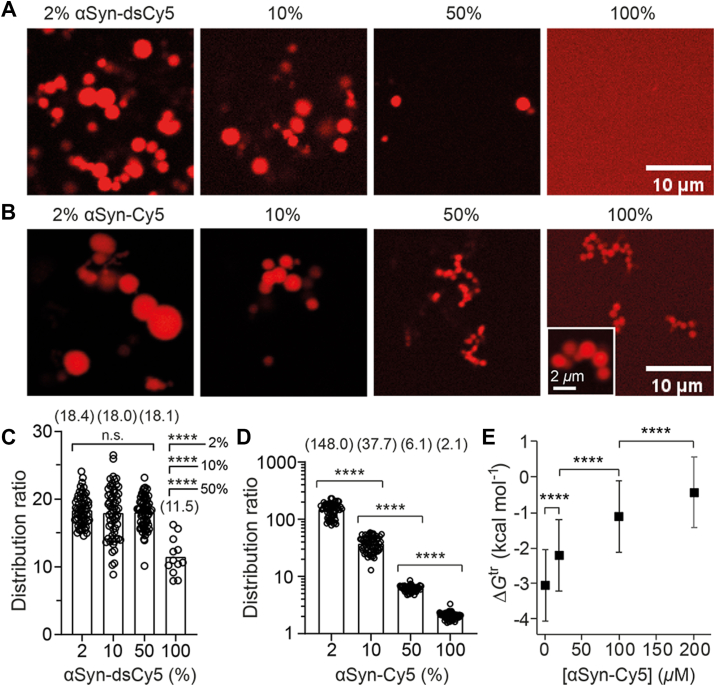
**Electrostatic effects on αSyn LLPS propensity.***A–B*, fluorescence images of αSyn LLPS samples prepared by mixing untagged αSyn with either (*A*) αSyn-dsCy5 or (*B*) αSyn-Cy5 at pH 7.4, acquired 1 day post-LLPS (d1). Samples contained varying proportions of labeled and unlabeled αSyn (indicated above images). *C–D*, distribution ratios of (*C*) αSyn-dsCy5 and (*D*) αSyn-Cy5 in LLPS samples with different mixing ratios (*n* = 12 for 100% αSyn-dsCy5; *n* = 60 for all other groups). Mean values were shown in parentheses. Welch and Brown-Forsythe ANOVA tests were performed on 2%, 10%, and 50% αSyn-dsCy5 in (*C*), and two-tailed *t* tests was used to compare 100% dsCy5 with the remaining groups in (*C*). Two-tailed *t* tests were performed pairwise on (*D*) (n.s. for *p* > 0.05, ∗∗∗∗ for *p* < 0.0001). *E*, mean Δ*G*^tr^ of αSyn-Cy5 calculated from the data in *D*. Error bars represent standard deviation. Statistical comparisons by pairwise two-tailed *t* tests. αSyn, α-synuclein; LLPS, liquid–liquid phase separation.

The homogeneous fluorescence intensity of dye-labeled αSyn (Fig. 1*B*) and freely diffusing dyes (Fig. 3, *A* and *C*) within the interior of the condensate’s cross section suggested a rather uniform distribution of charges. We hypothesized that αSyn LLPS is governed by a balance between self-association and unscreened negative charge repulsion, hence generating condensate with uniform charge density on the condensate’s length scale. To test this, we prepared LLPS samples with increasing proportion of dsCy5-labeled αSyn, simulating the addition of excess negative charges. Indeed, the number of droplets dropped drastically as the percentage of αSyn-dsCy5 increased (Fig. 4*A*). At 100% αSyn-dsCy5, no droplets formed after 90 min; by 1 day, only sparse droplets and irregular aggregates were observed, with lower distribution ratios than other mixtures (Fig. 4*C*). This confirmed that excess negative charge antagonizes αSyn LLPS under our conditions.

We then performed the same type of experiment using αSyn-Cy5, which carries an additional positive charge instead. In this case, formation of round-shaped droplets could be observed throughout the samples up to 100% of αSyn-Cy5, even though there was a clear trend of decreasing sizes (Fig. 4*B*). Apparently, the property of the condensate had been modified by the addition of αSyn-Cy5, as shown by the decreasing distribution ratio (Fig. 4*D*). This was in contrast to the experiment with increasing proportion of αSyn-dsCy5, in which distribution ratios remained essentially constant except for the samples with 100% αSyn-dsCy5 (Fig. 4*C*).

When Δ*G*^tr^ remained constant as the proportion of αSyn-dsCy5 was increased, it implies either LLPS is driven equally by αSyn:αSyn (homotypic) and αSyn:αSyn-dsCy5 (heterotypic) interactions (26) or αSyn-dsCy5 does not support LLPS at all. The near-complete LLPS inhibition at 100% αSyn-dsCy5 supports the latter scenario, indicating that αSyn-dsCy5 behaved like a “client” (35) in the condensate formed by untagged αSyn, thus αSyn-dsCy5 would have minimal effect on the phase-separated state of untagged αSyn.

On the other hand, αSyn-Cy5 acted as the “scaffold” (35) together with untagged αSyn to form condensates with variable distribution ratios depending on the ratio of their concentrations. Its Δ*G*^tr^ exhibited a concentration-dependent hyperbolic curve similar to the results on intracellular NPM1 condensate (26), showing that heterotypic αSyn:αSyn-Cy5 interaction is more stabilizing than homotypic αSyn-Cy5:αSyn-Cy5, and charge neutralization may play a role in their LLPS (Fig. 4*E*). These results indicated that LLPS propensity and the resulting phase-separated states of αSyn are highly sensitive to the mixture composition of αSyn modified by moiety of distinct net charges.

### Negatively charged αSyn condensates in the cell

**Figure 5:**
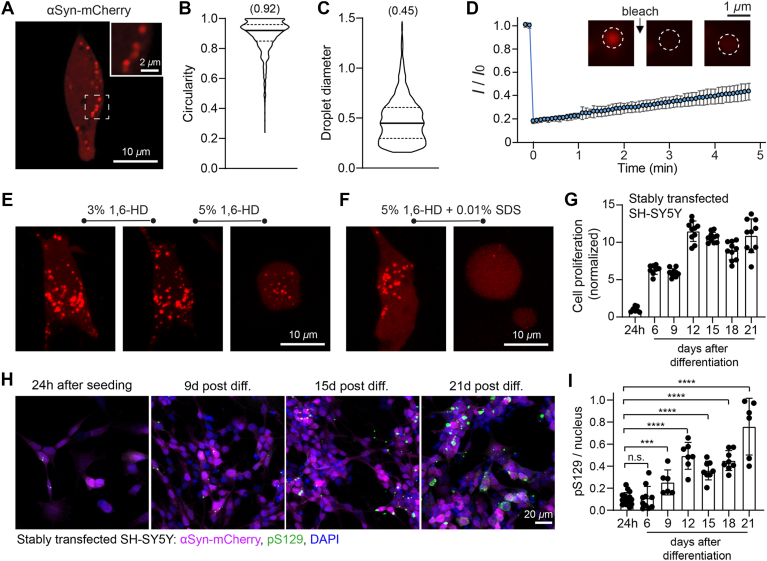
**Cellular model of αSyn LLPS.***A*, representative confocal image of differentiated SH-SY5Y cells overexpressing αSyn-mCherry (with mCherry fused *via* a flexible –GSGSGSGS– linker). *B–C*, quantification of condensate morphology from structured illumination microscopy (SIM) images of the cells overexpressing αSyn-mCherry (*n* = 1050 droplets from 12 F.O.V.). Median values are indicated in parentheses. *D*, fluorescence recovery kinetics of individual condensates monitored for 4.8 min postbleaching (mean ± s.d., *n* = 7 droplets). *E*–*F*, effects of treating αSyn-mCherry-overexpressing cells with: (*E*) 3% or 5% (w/v) 1,6-HD, (*F*) combined 5% (w/v) 1,6-HD, and 0.01% (w/v) SDS. (*G*). Cell proliferation assay measured by CCK8 in SH-SY5Y cells stably expressing αSyn-mCherry over a 21-days differentiation/maintenance period. Data: mean ± s.d. (*n* = 10 wells per group). *H*–*I*, immunocytochemistry analysis: quantification of αSyn-pS129-positive inclusions during the 21-day period. Data expressed as average inclusion count per nucleus per field of view [*n* = 16 (24 h), 10 (6 days), 6 (9 days), 7 (12 days), 8 (15 days), 8 (18 days), and 6 (21 days)]. Error bars: mean ± s.d. Statistical significance was assessed by two-tailed *t* tests (n.s. for *p* > 0.05, ∗∗∗ for *p* < 0.001, ∗∗∗∗ for *p* < 0.0001). 1,6-HD, 1,6-hexanediol; αSyn, α-synuclein; LLPS, liquid–liquid phase separation.

We went on to study LLPS of αSyn in the cell using different cell lines, some of which had been reported by other groups with disparate observations (13, 36, 37, 38). First, in COS-7 cells, neither the overexpression of αSyn alone nor its coexpression with synapsin-1 resulted in any observable αSyn droplets (data not shown). Second, in HEK293T cells, only a very small percentage of cotransfected cells exhibited αSyn droplet formation (Fig. S6). Conversely, a robust αSyn LLPS was achievable by overexpression of αSyn-mCherry in differentiated SH-SY5Y cells—a model of cholinergic/dopaminergic neuron for PD research (39) (Fig. 5*A*). Notably, these highly mobile αSyn droplets exhibited a spherical shape (Fig. 5*B*) with a rather uniform size of about 0.5 *μ*m (Fig. 5*C*) and were dispersed throughout the cytosol, unlike the observation of larger-diameter, perinuclear aggresome as reported by others (37).

Similar to *in vitro* αSyn condensates, these intracellular droplets displayed limited fluidity, as evidenced by fluorescence recovery after photobleaching analysis (Fig. 5*D*). Treatment of αSyn-mCherry-expressing cells with 3% (w/v) 1,6-HD had minimal impact on condensate morphology. However, at 5% (w/v) 1,6-HD, cells began to shrink and detach, suggesting cellular stress prior to any significant condensate disruption (Fig. 5*E*). Strikingly, only the combined treatment of 5% (w/v) 1,6-HD and 0.01% (w/v) SDS fully dispersed the condensates before cell detachment (Fig. 5*F*). The necessity of such harsh conditions—including a membrane-disrupting detergent—implies these condensates adopt a functional gel-like state, consistent with prior reports on P granules in *Caenorhabditis elegans* embryos (33) and chromatin (40). This resistance to 1,6-HD alone further distinguishes them from more dynamic liquid droplets.

Having established the gel-like state of intracellular αSyn condensates, we next investigated whether these condensates impair cellular health. We generated an SH-SY5Y cell line with stable genomic integration of αSyn-mCherry, which formed condensates upon differentiation. As expected, differentiation suppressed exponential cell proliferation, though limited growth persisted until stabilization at 12 days postdifferentiation (Fig. 5*G*). Notably, condensate formation did not induce overt cytotoxicity, suggesting these gel-like assemblies are not immediately detrimental to cell viability. Since phosphorylation at serine 129 (pS129) is a hallmark of pathological αSyn in PD and synucleinopathies, we assessed whether pS129-positive inclusions were present. Intriguingly, only low levels of pS129 were detected initially, with a gradual increase over the 21-days differentiation/maintenance period (Fig. 5, *H* and *I*). This time-dependent accumulation mirrors Lewy body maturation, implying our model captures late-stage transitions from condensates to fibrillar aggregates. Together, these results indicate that gel-like αSyn condensates are well-tolerated by differentiated SH-SY5Y cells in the short term.

**Figure 6:**
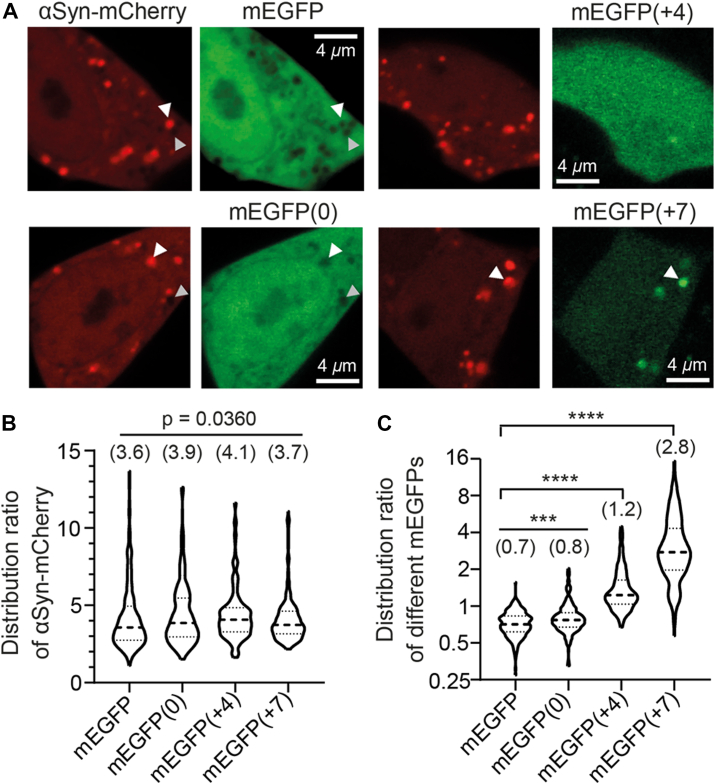
**Negatively charged αSyn condensates in cells.***A*, fluorescence images of SH-SY5Y cells coexpressing αSyn-mCherry and either wildtype or mutant mEGFP with varying net charges (indicated in brackets). For wildtype mEGFP and mEGFP(0), *white arrowheads* highlight examples of mEGFP exclusion from αSyn condensates, while *gray arrowheads* denote cavities excluding both αSyn-mCherry and mEGFP. For mEGFP(+7), *white arrowheads* indicate mEGFP enrichment within condensates. *B*–*C*, violin plots of the distribution ratio of (*B*) αSyn-mCherry and (*C*) different mEGFP(X) variants determined from the respective combination of co-expressed cells as shown in (*A*). For each combination, *n* = 230 from 20 cells in duplicate cultures. Median (in parentheses) and quartiles are marked by *dashed* and *dotted lines*, respectively. Kruskal–Wallis test was performed on data in (*B*), while Mann*–*Whitney test was performed on data in (*C*) (∗∗∗ for *p* < 0.001, ∗∗∗∗ for *p* < 0.0001). αSyn, α-synuclein.

To further assess whether αSyn condensates exhibit a negative electrostatic potential, we cotransfected SH-SY5Y cells with αSyn-mCherry and mEGFP variants of differing net charges (Fig. 6*A*), then quantified their distribution ratios using methods analogous to *in vitro* experiments. Wildtype mEGFP has a net charge of −8, while three other mEGFPs variants with a net charge of 0, +4, and +7, respectively, were generated based on the protein supercharging principle (41) (refer to Fig. S7 for amino acid sequences). Clearly, αSyn condensates appeared to partially exclude mEGFP and mEGFP(0), while mEGFP(+4) was distributed homogenously and mEGFP(+7) was enriched in the condensates (Fig. 6*A*). Quantification of their distribution ratios showed that wildtype mEGFP and mEGFP(0) had ratios < 1, mEGFP(+4) was near unity, and mEGFP(+7) showed 2.8-fold enrichment in condensates (Fig. 6*C*). The trend showed a positive correlation between mEGFP’s net charge and its partitioning, hence strongly supporting the existence of a negative electrostatic potential in αSyn condensates. In all cases, αSyn-mCherry exhibited only slight variation in distribution ratio (Fig. 6*B*), indicating that overexpressed mEGFP(X) acted as a “client” to αSyn condensate. Another interesting observation to take note was the cavities that appeared to exclude both αSyn-mCherry and mEGFP (Fig. 6*A*, gray arrowheads), which indicated a possible existence of other negatively charged condensates.

## Discussion

**Figure 7:**
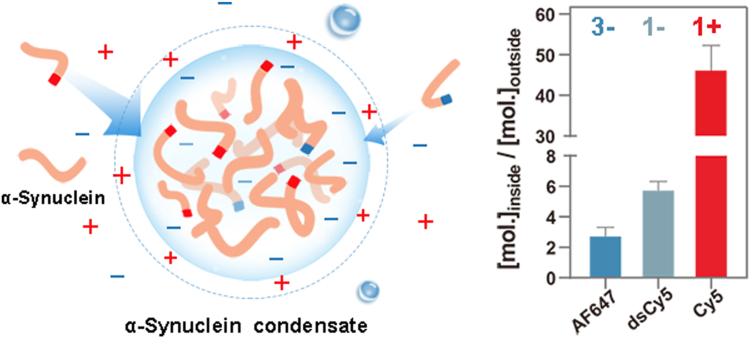
**Molecular partitioning behavior of α-synuclein condensate.** The formation of an electrostatic field that spans α-synuclein condensate modulates partitioning of molecules based on their net charges, hence providing opportunity for alternative control of biological activity and shedding light on the implication of aberrant partitioning of biomolecules in disease etiology. mol., molecular concentration; AF647, Alexa Fluor 647; dsCy5, sulfo-Cyanine 5.

In this study, we conducted a systematic analysis of the partitioning behavior of αSyn labeled with tags of varying net charges, revealing important methodological considerations for biomolecular LLPS research. First, our data pointed out the caveat of utilizing a charged fluorescent tag for quantifying the distribution ratio of a biomolecule upon LLPS, particularly for charged condensates (Fig. 7). As shown, αSyn condensate is a negatively charged simple coacervate, while FUS (simple coacervate) and PR_25_:PolyU (complex coacervate) were also reported to carry negative zeta potentials (22). These observations suggest that electrostatic potential may be a common feature of biomolecular condensates, underscoring the importance of carefully considering tag charge effects in LLPS experiments.

Second, our results further demonstrate how tag net charge influences the thermodynamics of αSyn LLPS, affecting both its propensity to phase separate and the resulting condensate properties. Remarkably, αSyn assumed distinct roles as either a "client" or "scaffold" depending on the charge characteristics of its conjugated tag, revealing electrostatics as a key determinant of these phase behaviors. In the case of αSyn-dsCy5, LLPS was completely inhibited, suggesting that repulsive electrostatic interactions dominated the system's Gibbs free energy landscape (42). This phenomenon parallels observations with TDP-43 fragments of varying isoelectric points (43), underscoring the general importance of charge balance in LLPS regulation. We propose this mechanism may explain why tetracysteine-tagged αSyn in HeLa cells required cation treatment (ammonium ferric citrate or copper sulfate) to undergo efficient LLPS (13)—the added cations likely neutralized excess negative charge on αSyn, thereby promoting phase separation.

Molecular partitioning is a critical feature of biomolecular condensates, as the selective enrichment or exclusion of specific biomolecules is a proposed functional outcome of LLPS in biological systems (44, 45). *Aberrant phase separation* typically refers to dysregulated LLPS that contributes to disease pathogenesis (46, 47), with most studies focusing on sol-gel transitions leading to pathogenic aggregate formation (48). However, emerging evidence highlights disease mechanisms involving *aberrant partitioning* of biomolecules that were also electrostatically driven (49, 50). For instance, a frameshift mutation in HMGB1 increases its positive charge, causing mispartitioning into the nucleolus and resulting in a rare developmental disorder (49). Notably, bioinformatic analyses suggest that similar nucleolar mispartitioning mechanisms may underlie other genetic diseases (49). In this study, we established a stably transfected SH-SY5Y cell line that forms αSyn condensates and progressively accumulates pS129. This model provides a valuable tool for investigating the physiological consequences of αSyn LLPS, including the sequestration of positively charged proteins like PINK1, and its potential role in PD beyond canonical amyloid fibril formation.

While the pharmaceutical industry routinely uses partition coefficients as indicators of drug hydrophobicity and membrane permeability (51), effective drug targeting requires additional considerations—particularly intracellular localization. This principle was elegantly demonstrated by recent work showing how differential partitioning of cancer drugs into specific biomolecular condensates determines their therapeutic efficacy (52). Our findings reveal that condensate electrostatic potential dramatically influences small molecule partitioning. In αSyn condensates, Cy5-maleimide showed 10-fold greater enrichment than its structural analog dsCy5-maleimide, despite only a 2*e* charge difference. While specific binding accounts for much of this effect, electrostatic interactions alone would predict a 2-fold enrichment difference. These results highlight the need for further studies to develop general models relating molecular charge to condensate enrichment levels and to investigate how condensate electrostatic potential might affect molecule-protein binding equilibria.

In conclusion, we have demonstrated that αSyn condensates, formed either *in vitro* or in cells, maintain a negative electrostatic potential that spans the cross section of the condensate. This property actively modulates molecular partitioning based on net charge. Furthermore, we showed that the net charge of fluorescent tags significantly influences αSyn LLPS behavior. These findings underscore the need to systematically evaluate electrostatic potentials in diverse biomolecular condensates and their functional consequences for both phase separation propensity and molecular partitioning.

## Experimental procedures

### Materials

Cyanine dyes were purchased from the respective companies: Alexa Fluor 647 C2 maleimide (Catalog No. A20347, Thermo Fisher Scientific), diSulfo-Cy5 maleimide (Catalog No. BDC-47, CAS No. 2130955-10-9, CONFLUORE), and Cy5 maleimide non-sulfonated (Catalog No. A8139, APExBIO). Riboflavin (CAS No. 83-88-5), riboflavin-5′-phosphate sodium salt dehydrate (riboflavin-PO4, CAS No. 6184-17-4), NaOH (CAS No. 1310-73-2), Tris-HCl (CAS No. 1185-53-1), NaCl (CAS No. 7647-14-5), MES (CAS No. 145224-94-8), and EDTA (CAS No. 60-00-4) were all purchased from Sigma. PEG-8000 (CAS No. 25322-68-3) was purchased from Solarbio. IPTG (Catalog No. ST098, CAS No. 367-93-1), DTT (Catalog No. ST043, CAS No. 3483-12-3), PMSF (Catalog No. ST505, CAS No. 329-98-6), Hepes (Catalog No. ST092, CAS No. 7365-45-9), and TCEP (Catalog No. ST045, CAS No. 51805-45-9) were all purchased from Beyotime. The reagents used for cell culture and transfection were from Beyotime, Gibco, and Thermo Fisher Scientific, unless otherwise specified, including brain-derived neurotrophic factor (BDNF, Catalog No. 450-02-10, PEPROTECH) and EC23 (Catalog No. HY-12309 EC23, CAS No. 2130955-10-9, MedChemEpress). All solutions were freshly prepared and filtered through a 0.22 *μ*m syringe filter for each use.

### Protein expression and purification

Expression and purification of human full-length αSyn, αSyn(A140C), and αSyn(1-103) were performed by a combination of purification steps (53). Briefly, colonies of *Escherichia coli* BL21(DE3) transformed with the respective expression vectors were cultivated in LB broth (100 mg/L ampicillin) at 37 °C with shaking (220 rpm). At *A*_600_ of 0.6 to 0.8, the cultures were induced with 1 mM IPTG for 4 h. The cells were then harvested by centrifugation (10,000 rpm, 10 min), and the pellet was kept frozen at −80 °C. For protein purification, the pellet was resuspended in lysis buffer (10 mM Tris-HCl, pH 8.0, 1 mM EDTA, 1 mM DTT, and 1 mM PMSF) and sonicated at 300 W for 20 min. Cell debris was then separated by centrifugation (10,000 rpm, 20 min), and the supernatant was added 1.5% HCl to pH 3.5, followed by centrifugation (10,000 rpm, 10 min) to remove unwanted proteins. The supernatant was added 1 M NaOH to pH 7.5. Then, 8.7 g ammonium sulfate was added for αSyn to precipitate at 4 °C for at least 1 h. Subsequently, the solution was centrifuged at 10,000 rpm and 4 °C for 20 min to pellet down αSyn. The pellet was solubilized and dialyzed against the buffer (20 mM Tris-HCl, pH 8.0, 1 mM EDTA, and 150 mM NaCl) overnight at 4 °C using a 10 kDa MWCO mini-dialysis unit (Catalog No. MD34-10KD, RuiTaibio). This process was carried out to remove salts. Finally, the protein solution was loaded onto HiLoad 26/600 Superdex 200 pg column attached to an AKTA purifier (GE Healthcare) and eluted isocratically at 4 °C in the buffer (25 mM Hepes, pH 7.4, 150 mM NaCl). The monomeric αSyn fraction was either lyophilized and redissolved or directly exchanged using centrifugal concentrator (3000 MWCO, Catalog No. VS2021, Sartorius) into two different buffers: (i) 25 mM Hepes, 150 mM NaCl, pH 7.4 or (ii) 25 mM MES, 150 mM NaCl, pH 5.5 and then aliquoted, flash frozen, and stored at −80 °C for future use. Purity of the protein was confirmed by running sodium dodecyl sulfate-polyamide gel electrophoresis (Beyotime).

### Fluorophore labeling of αSyn

The maleimide dyes (AF647, dsCy5, and Cy5) were dissolved in DMSO at the concentration of 10 mM. Eight- to 10-fold molar excess of the individual dye was added to 100 *μ*M αSyn(A140C) protein (TCEP pretreated for 30 min) and incubated at room temperature for 1 h. The reaction mixture was then diluted with 1 ml buffer (25 mM Hepes, pH 7.4, 150 mM NaCl) and dialyzed against the same buffer overnight at 4 °C using a 10 kDa MWCO mini-dialysis unit (Catalog No. MD34-10KD, RuiTaibio) to remove unreacted dye. The extent of modification of the protein with the dye was determined by NanoDrop One^C^ (Thermo Fisher) and SDS-PAGE. For another experiment condition (25 mM MES, pH 5.5, and 150 mM NaCl), the dye-labeled αSyn(A140C) protein was dialyzed against the target buffer overnight at 4 °C and concentrated by 3000 MWCO Protein Concentrators PES (Catalog No. VS2021, Sartorius). All labeled proteins were confirmed by mass spectrometry (Figs. S8–S12). High-resolution mass spectrometry (ESI) spectra were obtained using a Thermo Fisher Q-Exactive Mass Spectrometer. 0.1 mg/ml proteins were measured in 25 mM Hepes, pH 7.4, and 150 mM NaCl.

### Circular dichroism spectroscopy

30 *μ*M αSyn, AF647-, dsCy5-, and Cy5-labeled αSyn at 50 mM sodium phosphate buffer, pH 7.4, were performed *via* Applied Photophysics Chirascan circular dichroism spectrometer. Spectra were recorded from 260 to 200 nm at 25 °C with a speed of 100 nm/min *via* a circular dichroism micro cuvette of 1 cm path length.

### *In vitro* LLPS assay of αSyn

The protein solution was thawed and precleared by centrifugation (10,000 rpm, 10 min) at 4 °C, and its final concentration was determined. The LLPS sample was prepared by mixing the components accordingly, with 10% (w/v) PEG-8000 being added at the end and mixed gently to ensure homogeneity. Ten microliters of the mixture was drop-casted in the middle of a 35-mm glass bottom microwell dish (Catalog No. P35GC-1.0-14-C, MatTek Corporation), air-exposed on the surface of heat plate (37 °C for 5 min), and then covered with a coverslip and sealed with UV-LED resin (see Fig. S2). The dish was then kept at 37 °C for the whole duration of our experiment including microscopy.

### Confocal microscopy

Fluorescence images were acquired on a Zeiss LSM 900 inverted confocal microscope (Carl Zeiss AG) with a Plan-Apochromat 63×/1.40 Oil DIC M27 objective lens. The sample dishes were kept inside a temperature-regulated chamber (37 °C, 5% CO_2_, STXG-WSKMX-SET, Tokai Hit Co., Ltd) mounted on the microscope stage. Supply of CO_2_ was not used for *in vitro* LLPS samples. Cyanine dyes were excited by 653-nm laser and detected at 656 to 700 nm, riboflavin/riboflavin-PO_4_ were excited by 488-nm laser and detected at 410 to 561 nm, mEGFP/EGFP were excited by 488-nm laser and detected at 400 to 560 nm, and mCherry was excited by 561-nm laser and detected at 576 to 700 nm. Laser power was adjusted to avoid saturation of fluorescence intensity. For *in vitro* LLPS experiment, 10 images (20.28 × 20.28 *μ*m, 512 × 512 pixels, 16 bit-depth) were acquired for each time point for each sample. For imaging the SH-SY5Y cells, 1 *μ*m-separated z-stack of images (pixel size = 0.085 × 0.085 *μ*m, 16 bit-depth) were acquired for each cell.

### Fluorescence recovery after photobleaching

For *in vitro* condensates, imaging (0.1% laser power) and bleaching (100% laser power) were performed using a 639 nm laser. A z-stack of seven slices (total depth: 3 μm, 173 × 173 pixels, pixel size = 0.111 μm) was acquired every 5 s to account for droplet movement. The same imaging mode was used for intracellular condensates, except with a 543 nm excitation laser.

### Structured illumination microscopy

Live structured illumination microscopy imaging was performed at 37 °C using a Multi-SIM system (NanoInsights-Tech Co., Ltd) with a stage-top incubator (Okolab H301). Z-stack images of SH-SY5Y cells were acquired with 1536 × 1536 pixel fields (pixel size = 31.3 nm), a 0.135 μm inter-stack interval, and a total z-axis range of 1.5 μm.

### Image analysis

All images were analyzed using ImageJ software (National Institutes of Health). To determine the distribution ratio, which is the ratio of the mean fluorescence intensity between inside and outside of the droplet, pairs of region of interest (0.79 *μ*m diameter circle) were chosen such that one was centered in the droplet and the other was in the vicinity outside the droplet. The brighter droplets were chosen in order to obtain intensity closer to the center of the droplet. For the distribution ratio in SH-SY5Y cells, circular region of interests with 0.34 *μ*m diameter were chosen.

### Zeta potential

Measurement of zeta potential was performed using Zetasizer Nano ZS (Malvern Instruments Ltd) with disposable folded capillary cell (DTS1070, Malvern Panalytical Ltd) and analyzed using Zetasizer Software (Malvern Panalytical Ltd). To acquire large volume of LLPS samples for the measurement, 1.5 ml solution of 200 *μ*M αSyn containing 10% (w/v) PEG-8000 was drop-casted onto a 35-mm glass bottom microwell dish and incubated for 30 min at 37 °C, aspirated, and placed inside an Eppendorf tube for incubation of another 2 h at 37 °C. The final volume of the solution was about 1.3 ml, and droplet formation was confirmed by confocal microscopy before zeta potential measurement. Zeta potential was measured at 37 °C three times for each sample. To calculate zeta potential, the parameters for water as the dispersant at 37 °C were used, *i.e.* viscosity = 0.6864 cP and dielectric constant = 74.4. The output zeta potential distribution was interpolated and fitted to different Gaussian mixture models using MATLAB (The MathWorks, Inc.).

### Isothermal titration calorimetry

The thermodynamic parameters of the fluorophores binding to αSyn protein were measured using a Nano ITC200 instrument from TA Instruments. Experiments were carried out at 25 °C in buffer (25 mM Hepes, pH 7.4, 150 mM NaCl) with 10% (v/v) DMSO. All measurements were performed two times, and all solutions were accurately degassed before the ITC experiments to avoid the formation of air bubbles in the calorimeter vessel. The concentration of AF647-, dsCy5-, Cy5-maleimide, and riboflavin was 1 mM and titrated into αSyn protein solution of 0.1 mM. The concentration of riboflavin-5′-phosphate was 0.5 mM and titrated into αSyn protein solution of 0.02 mM. Twenty-five injections of 2 *μ*l fluorophore solution were added into a 200-*μ*l cell containing αSyn protein solution, every 250 s. All the measurements were conducted at a continuous stirring rate of 350 rpm. Control experiments were carried out to calculate the heat of dilution. Integrated heat data obtained from the titrations were fitted to the independent model using NanoAnalyze software (TA Instruments) as well as a ITC Data Fitting Program written by Debashish Sahu (https://sites.lsa.umich.edu/debsahu/2016/04/27/itc-data-fitting-program/).

### Growth and differentiation of SH-SY5Y cells

The obtained SH-SY5Y cells had been tested for *mycoplasma*. Differentiation of SH-SY5Y cells was performed according to Förster *et al.* (54) with minor modifications. Cells were maintained at 37 °C and 5% CO_2_ in Dulbecco’s Modified Eagle’s Medium (DMEM, Gibco) supplemented with 10% fetal bovine serum (FBS, Thermo Fisher) and 1% penicillin/streptomycin (BI). T25 cell culture flasks (Sarstedt) were used for cell proliferation. After cells had reached 80 to 90% confluence, splitting was done by washing cells with phosphate-buffered saline (Beyotime) once and incubating them with trypsin-EDTA for 2 min at 37 °C. The cell pellet was then resuspended in DMEM medium (10% FBS, 1% pen/strep) and plated in μ-Slide 4 Well Chamber Slide (Catalog No. 80426, tissue culture-treated, ibidi GmbH). After 24 h, culture medium was switched to DMEM/F12 (5% FBS) supplemented with 10 *μ*M all-trans-retinoic acid (EC23) to promote differentiation and induce neuronal phenotype. Three days later, culture medium was switched to Neurobasal medium (containing B27 supplement and GlutaMAX) supplemented with 50 ng/ml of BDNF. Afterward, culture medium was replaced every 3 days using the same recipe, *i.e.* NBA medium with B27, GlutaMAX, and BDNF, to maintain the differentiated cells. Successful differentiation of SH-SY5Y cells was monitored by morphological assessment of neurite outgrowth.

Fluorescent protein (either mEGFP or mCherry) was fused to the C terminus of human αSyn following four repeats of glycine and serine, *i.e.* αSyn-(GS)4-FP. The respective DNA constructs were cloned into pcDNA3.1(+) vector with a CMV promoter. Transfection of SH-SY5Y cells with the respective αSyn expression vectors was performed using Lipofectamine 6000 (Thermo Fischer) following manufacturer's instructions. Briefly, DNA (2.4 *μ*g in total) was incubated with Lipofectamine 6000 (5 *μ*l) and Opti-MEM (120 *μ*l) for 10 min at room temperature and then added into the cells with ∼700 *μ*l culture medium. The cells were incubated for 4 to 6 h (37 °C and 5% CO_2_), and the culture medium was then replaced by fresh medium.

### Generation of stably transfected SH-SY5Y cell line

A stable cell line was generated *via* lentivirus-mediated infection. αSyn-mCherry was cloned into pHBLV-CMV-MCS-IRES-Puro vector and was packaged into the lentivirus using the third generation technology (Guangzhou PackGene Biotechnology Co, Ltd). Cells were seeded in 24-well plates and infected with lentivirus the next day. After 24 h, puromycin (2.5ug/ml) was used for screening. The culture medium was changed every 3 days for three rounds. Then, cells were transferred to T25 flasks with puromycin-containing medium. After three more screenings, puromycin was discontinued.

### Cell viability assay

Cell viability at different growth stages was assessed using the Cell Counting Kit-8 (CCK-8) assay. Briefly, culture medium in each well of the 96-well plate was replaced with 100 μl fresh medium containing 5 μl CCK-8 solution. Following 2 h of incubation at 37 °C with 5% CO_2_, absorbance was measured at 450 nm using a BioTek SH1M2 microplate reader (Shenzhen Boxing Biotechnology Co, Ltd).

### Immunocytochemistry and imaging

To examine αSyn phosphorylation dynamics across cell growth stages, immunocytochemistry was performed at designated time points. Cells were stained with anti-pS129 primary antibody (1:1000, BioLegend #825701) and counterstained with DAPI (1:2000, Coolaber #CD4261) for nuclear visualization. Primary antibodies were detected using appropriate fluorescently labeled secondary antibodies. Confocal imaging was conducted on a Zeiss LSM 900 inverted microscope equipped with a Plan-Neofluar 20 × /0.50 M27 objective. Fluorescence signals were acquired using the following parameters: pS129: 488 nm excitation, 400 to 575 nm emission collection; DAPI: 405 nm excitation, and 400 to 605 nm emission collection. Z-stack images (319.45 × 319.45 μm, 512 × 512 pixels, 8 bit depth) were acquired with layer numbers adjusted per sample requirements. pS129 signal quantification was performed using Imaris software (Bitplane), while nuclear analysis (DAPI) was conducted using ImageJ (NIH).

## Data availability

Original data are available from the corresponding author (L. E. W.) upon request.

## Supporting information

This article contains supporting information.

## Conflict of interest

The authors declare that they have no conflicts of interest with the contents of this article.
