# Supplementary material for: Negatively charged α-synuclein condensate modulates partitioning of molecules
Source: J Biol Chem. 2025 Jul 26;301(9):110530. doi: 10.1016/j.jbc.2025.110530 (PMC12446775; doi:10.1016/j.jbc.2025.110530)
Supplement: Supporting Figures [file mmc1.pdf]

Supporting Information for:

Negatively charged α-Synuclein condensate modulates partitioning of molecules

Qingqing Yang[1,2#], Shunfa Chen[1,2#], Pengfei Zhang[3], Zhonghua Lu[1,2], Shuwen Chang[4,5], Leo E. Wong[1,2*]

1 Institute of Biomedical and Health Engineering, Shenzhen Institute of Advanced Technology, Chinese Academy of Sciences, Shenzhen 518055, China

2 Shenzhen Key Laboratory for Molecular Biology of Neural Development, Shenzhen Technological Research Center for Primate Translational Medicine, Shenzhen-Hong Kong Institute of Brain Science, Shenzhen Institute of Advanced Technology, Chinese Academy of Sciences, Shenzhen 518055, China

3 Guangdong Key Laboratory of Nanomedicine, CAS-HK Joint Lab of Biomaterials, CAS Key Laboratory of Biomedical Imaging Science and System, Shenzhen Engineering Laboratory of Nanomedicine and Nanoformulations, CAS Key Lab for Health Informatics, Shenzhen Institute of Advanced Technology, Chinese Academy of Sciences, Shenzhen 518055, China

4 Institute of Brain Cognition and Brain Disease Institute, Shenzhen Institute of Advanced Technology, Chinese Academy of Sciences, Shenzhen 518055, China

5 Shenzhen Neher Neural Plasticity Laboratory, Shenzhen-Hong Kong Institute of Brain Science-Shenzhen Fundamental Research Institutions, Shenzhen 518055, China

*Corresponding author: leo.wong@siat.ac.cn

#These authors contributed equally to the work.

Supporting Figures:

Circular dichroism spectra of native and cyanine-labeled αSyn

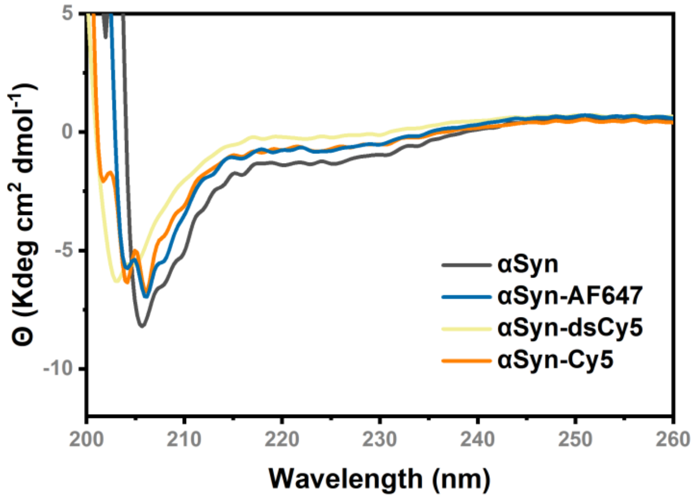

Figure S1. CD spectra of 30 μM αSyn, AF647-, dsCy5-, and Cy5-labeled αSyn at 50 mM sodium phosphate buffer, pH 7.4.

Preparation of αSyn LLPS samples

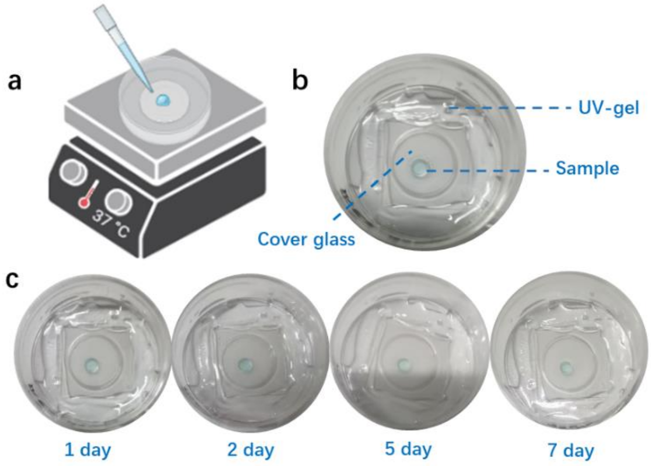

Figure S2. (a) Schematic depicting the preparation of αSyn LLPS sample. (b) Photo of a αSyn LLPS sample sealed in the 35-mm glass bottom dish immediately after preparation. (c) A series of photos were taken on the αSyn LLPS sample shown in (b) throughout the duration of the experiment from day 1 to 7, illustrating controlled volatilization and negligible shrinkage of the droplet.

Net charge of αSyn as a function of pH

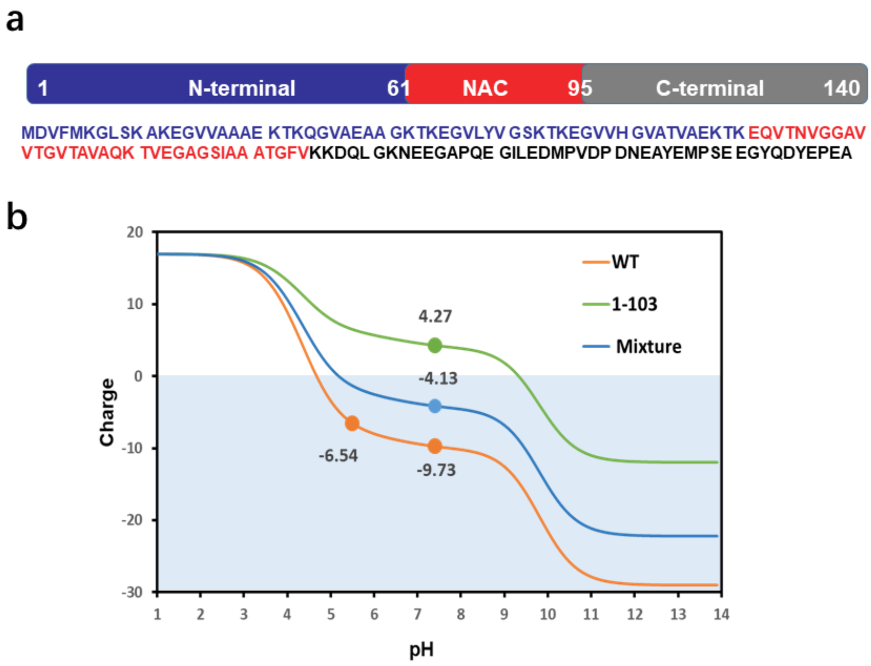

Figure S3. (a) Domain architecture of αSyn. The N-terminus (blue), non-amyloid-β component (NAC) (red) and C-terminus (grey) are shown. (b) The net charge as a function of pH, as predicted by Prot pi (https://www.protpi.ch/Calculator/ProteinTool), were plotted for full-length αSyn (WT, orange line), truncated αSyn(1-103) (1-103, green line), and a mixture of full-length αSyn and αSyn(1-103) at the molar ratio of [WT]:[1-103] = 0.6:0.4 (Mixture, blue line).

Gel-like properties of in vitro αSyn condensates

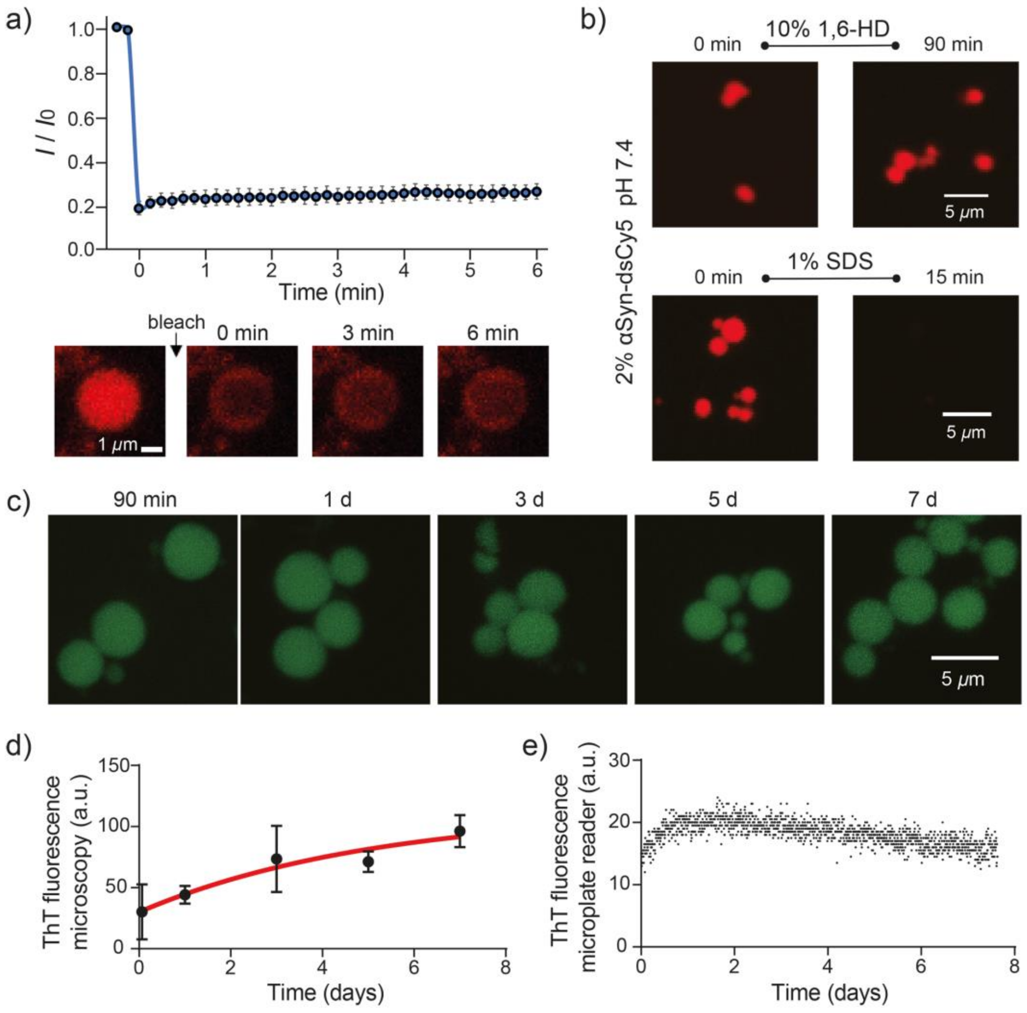

Figure S4. (a) FRAP kinetics of individual αSyn condensates (formed at pH 7.4, doped with 2% αSyn-dsCy5) monitored for 6 min post-bleaching. Data represent mean ± s.d. (n = 9 droplets; time window: 30–90 min post-LLPS). (b) Dispersal of in vitro αSyn condensates by treatment with 10% (w/v) 1,6-HD (upper panel) and 1% (w/v) SDS (lower panel). (c-d) Thioflavin T (ThT) fluorescence kinetics of αSyn LLPS samples (200 μM αSyn, 50 μM ThT in 25 mM HEPES, 150 mM NaCl, 10% PEG-8000, pH 7.4) monitored from 90 min to 7 days post-LLPS. Data represent mean ± s.d. (n = 48-52 droplets per experimental group). (e) Continuous ThT fluorescence measurements of quiescent LLPS samples acquired using a microplate reader (96-well plate format) over a 7-day observation period. Based on previous experience, fibril formation under shaking conditions typically produces signals several orders of magnitude stronger.

Zeta potential of PEG

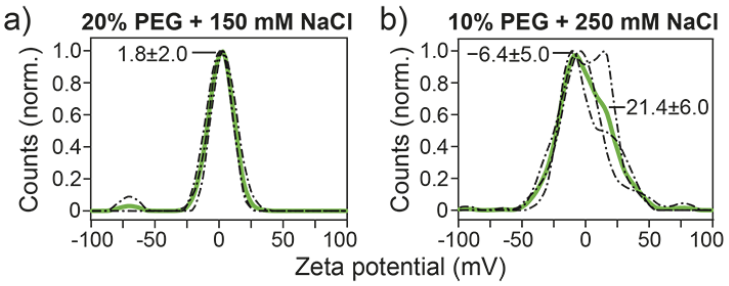

Figure S5. Distribution of the zeta potential measured on (a) 20% PEG with 25 mM HEPES, pH 7.4, 150 mM NaCl and (b) 10% PEG with 25 mM HEPES, pH 7.4, 250 mM NaCl.

Table S1. Suspension conductivity and electrophoretic mobility values of the zeta potential measurements.

Conductivity (mS/cm)			
10% PEG	14.8 ± 0.9		
10% PEG + 40 μM αSyn	15.9 ± 0.7		
LLPS pH 7.4	24.0 ± 1.3		
LLPS pH 5.5	27.0 ± 0.5		
LLPS pH 7.4 + αSyn(1-103)	20.3 ± 1.3		
Electrophoretic mobility (μm·cm/V·s)			
10% PEG	−0.3 ± 0.4		
10% PEG + 40 μM αSyn	−0.6 ± 0.1		
LLPS pH 7.4	−2.3 ± 0.5	−0.03 ± 0.19	
LLPS pH 5.5	−3.8 ± 0.3	0.3 ± 0.2	3.3 ± 2.2
LLPS pH 7.4 + αSyn(1-103)	−1.4 ± 0.5	0.7 ± 0.4	

Co-expression of αSyn and Synapsin-1 in HEK293T cells

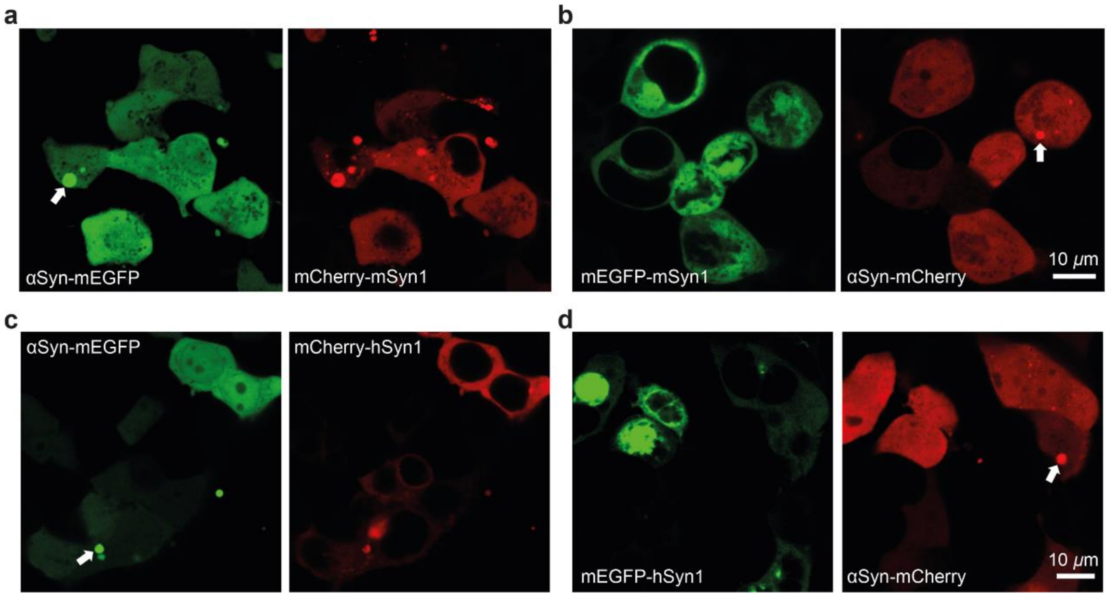

Figure S6. Representative fluorescence images of HEK293T cells co-expressing (a) αSyn-mEGFP and mCherry-mSyn1, (b) αSyn-mCherry and mEGFP-mSyn1, (c) αSyn-mEGFP and mCherry-hSyn1, and (d) αSyn-mCherry and mEGFP-hSyn1. Either mEGFP or mCherry was fused to the N-terminus of mouse Synapsin-1 and human Synapsin-1 with a linker of 10 and 14 amino acids, respectively, i.e. FP-LEVLFQGPGS-mSyn1 and FP-LEVLFQGPGSKLAT-hSyn1. All DNA constructs were cloned into pcDNA3.1(+) vector with a CMV promoter.

Amino acid sequences of mEGFP(X)

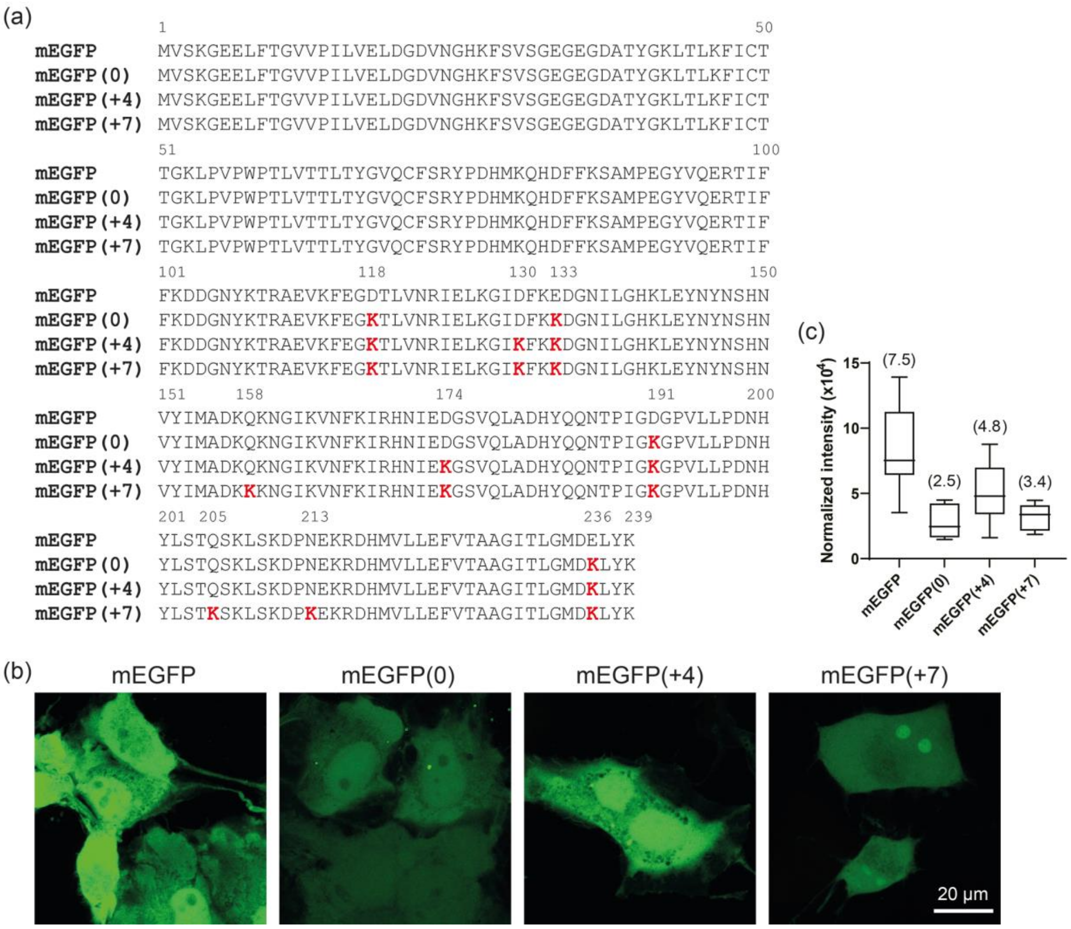

Figure S7. (a) Multiple sequence alignment of the members of wild-type mEGFP and three other mutant mEGFPs with a net charge of 0, +4, and +7, respectively. Residues in the mutant mEGFP that are different from wild-type mEGFP are colored in red. (b) Fluorescence images of COS7 cells overexpressing wild-type mEGFP and three mutant mEGFPs. (c) Box and whisker plot of the normalized fluorescence intensity determined from the respective cells in (b). Median is indicated in parentheses.

Mass spectrometry

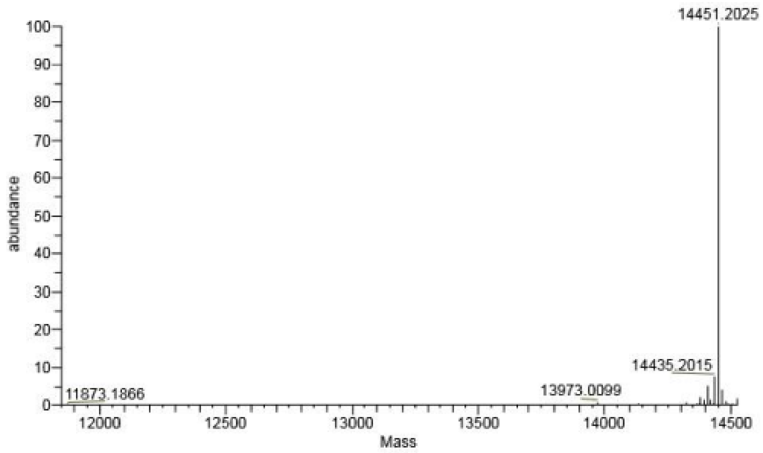

Figure S8. The ESI-MS spectrum of WT αSyn. Theoretical mass 14460.16, experimental mass 14451.20.

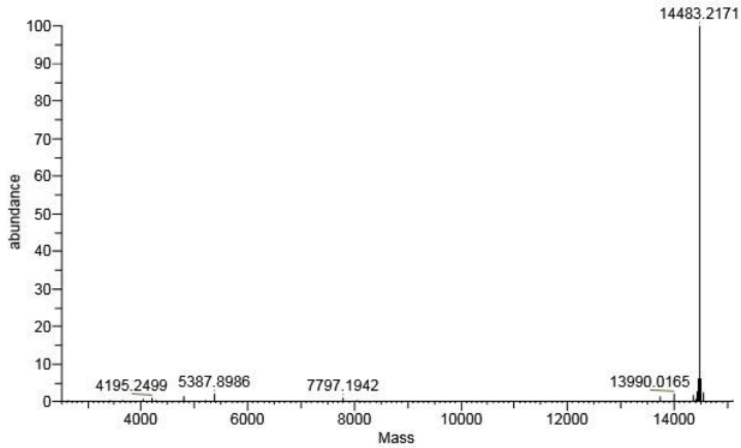

Figure S9. The ESI-MS spectrum of αSyn-140C. Theoretical mass 14492.13, experimental mass 14483.2171.

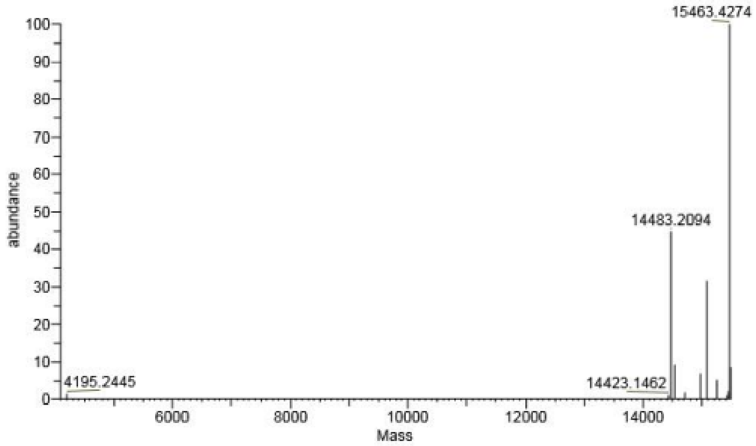

Figure S10. The ESI-MS spectrum of αSyn-AF647. Theoretical mass 15484.35, experimental mass 15463.42.

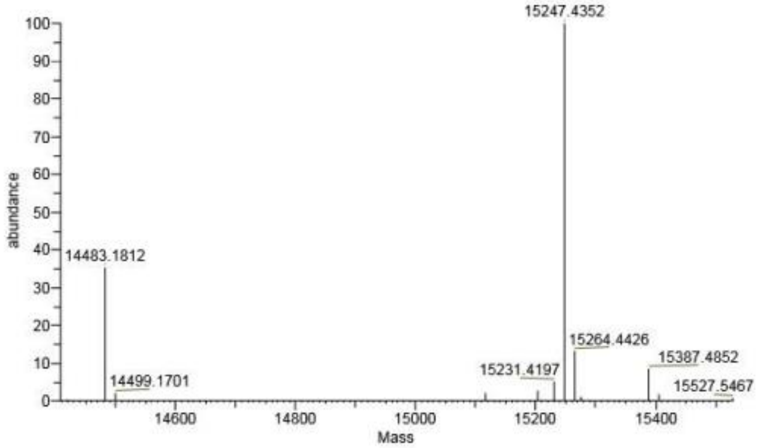

Figure S11. The ESI-MS spectrum of αSyn-dsCy5. Theoretical mass 15255.38, experimental mass 15247.44.

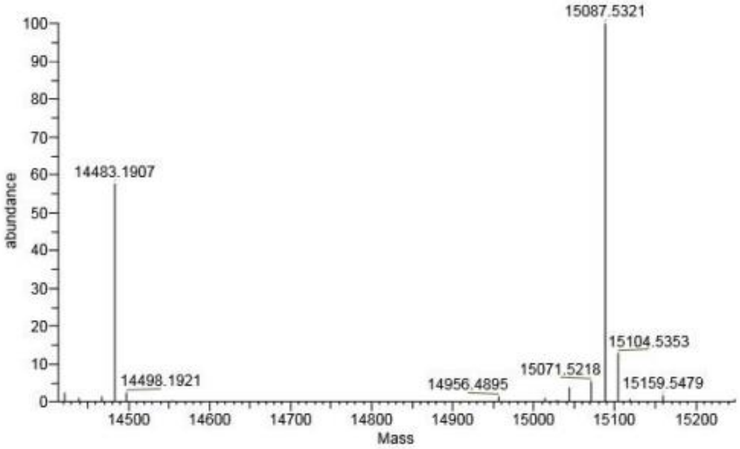

Figure S12. The ESI-MS spectrum of αSyn-Cy5. Theoretical mass 15097.48, experimental mass 15087.53.
